# Registered report: Kinase-dead BRAF and oncogenic RAS cooperate to drive tumor progression through CRAF

**DOI:** 10.7554/eLife.11999

**Published:** 2016-02-17

**Authors:** Ajay Bhargava, Madan Anant, Hildegard Mack, Reproducibility Project: Cancer Biology, Elizabeth Iorns

**Affiliations:** Science Exchange, Palo Alto, United States; Science Exchange, Palo Alto, United States; Science Exchange, Palo Alto, United States; Science Exchange, Palo Alto, United States; Science Exchange, Palo Alto, United States; Center for Open Science, Charlottesville, United States; 1 Shakti BioResearch LLC, Woodbridge, United States; 2 University of California, San Francisco, San Francisco, United States; Howard Hughes Medical Institute & University of Massachusetts Medical School, United States

**Keywords:** methodology, Reproducibility Project: Cancer Biology, B-RAF, K-RAS, melanoma, Human

## Abstract

The Reproducibility Project: Cancer Biology seeks to address growing concerns about reproducibility in scientific research by conducting replications of selected experiments from a number of high-profile papers in the field of cancer biology. The papers, which were published between 2010 and 2012, were selected on the basis of citations and Altmetric scores (Errington et al., 2014). This Registered Report describes the proposed replication plan of key experiments from "Kinase-dead BRAF and oncogenic RAS cooperate to drive tumor progression through CRAF" by Heidorn and colleagues, published in *Cell* in 2010 (Heidorn et al., 2010). The experiments to be replicated are those reported in Figures 1A, 1B, 3A, 3B, and 4D. Heidorn and colleagues report that paradoxical activation of the RAF-RAS-MEK-ERK pathway by BRAF inhibitors when applied to BRAF^WT^ cells is a result of BRAF/CRAF heterodimer formation upon inactivation of BRAF kinase activity, and occurs only in the context of active RAS. The Reproducibility Project: Cancer Biology is a collaboration between the Center for Open Science and Science Exchange, and the results of the replications will be published by *eLife*.

**DOI:** http://dx.doi.org/10.7554/eLife.11999.001

## Introduction

The RAS-RAF-MEK-ERK signaling pathway is routinely disregulated in many forms of cancer. Activating mutations in BRAF are found in almost half of all melanomas, and of these mutations, almost 90% involve a valine to glutamic acid transition at position 600 (BRAF^V600E^) (Solit and Rosen 2014). The therapeutic effect of drugs that target this form of BRAF have proved less efficacious than expected, due to an unexpected effect in cells that are BRAF^WT^; in these cells, drugs that target BRAF paradoxically activate rather than repress downstream signaling (Hall-Jackson et al., 1999a; Hall-Jackson et al., 1999b). In their 2010 paper, Heidorn and colleagues examined the mechanism of action behind this paradoxical activation of MEK/ERK signaling. Heidorn and colleagues first observed that paradoxical activation occurred only in the context of BRAF^WT^ and activated RAS, an observation confirmed by two other groups (Hatzivassiliou et al., 2010; Poulikakos et al., 2010). Dissecting the mechanism, they reported that the formation of BRAF/CRAF heterodimers was necessary for pathway activation, and formation of those heterodimers required active RAS signaling.

In Figure 1A, Heidorn and colleagues examined pathway activation in response to a range of drugs. The inhibitors, sorafenib, which targets and represses both BRAF and CRAF, PLX4720, which is highly selective for and inhibits the activity of BRAF^V600E^. 885-A, which specifically targets and inhibits BRAF, and the MEK inhibitor PC184352 were examined. As expected, all four drugs blocked MEK/ERK activation in BRAF^V600E^ A375 cells. However, in cells with active RAS, such as D04 cells (BRAF^WT^/NRAS^Q61L^), MEK/ERK signaling was not repressed by PLX4720 or 885-A. This paradoxical activation in BRAF^WT^ cells was also observed by several other groups (Carnahan et al., 2010; Joseph et al., 2010; Lee et al., 2010; Kaplan et al., 2011). This experiment will be replicated in Protocol 1.

Previous work had shown that activated RAS in melanoma signals through CRAF, while normal signaling in healthy melanocytes is accomplished through BRAF (Dumaz et al., 2006). To determine if CRAF was required for paradoxical pathway activation, Heidorn and colleagues treated D04 cells with siRNAs targeting NRAS and CRAF. Knockdown of either NRAS or CRAF abrogated activation of MEK/ERK by 885-A, as seen in Figure 1B. This experiment will be replicated in Protocol 2. The necessity of CRAF also explains the lack of activation upon treatment with sorafenib observed in Figure 1A; since sorafenib inhibits both BRAF and CRAF, it does not result in pathway activation.

Since activated RAS is known to drive heterodimerization of BRAF and CRAF (Weber et al., 2001), Heidorn and colleagues also tested if drug binding drove heterodimerization of BRAF and CRAF, and if this heterodimerization was dependent on active RAS signaling. In Figure 3A, they transfected D04 cells with a mutant version of CRAF that was unable to bind to RAS (CRAF^R89L^). Immunoprecipitation experiments showed that while CRAF^WT^ was able to bind to BRAF in the presence of activated RAS, CRAF^R89L^ was unable to bind to BRAF. This key experiment will be replicated in Protocol 3.

The authors showed that BRAF binds to CRAF but only in the presence of WT RAS, not oncogenic RAS. In Figure 3B, myc-tagged BRAF or myc-tagged mutant BRAF (^R188L^BRAF) were transfected into D04 cells and treated with either DMSO(-) or 885-A(+). The authors show that mutant of BRAF (^R188L^BRAF) does not bind to CRAF even in the presence of 885-A, which induces RAS activity.

After confirming that drug binding to BRAF drove BRAF binding to CRAF, Heidorn and colleagues tested a kinase dead version of BRAF (BRAF^D594A^) (Figure 4D). Interestingly, this version of BRAF still bound to CRAF, indicating that it is not drug binding per se, but inhibition of BRAF activity, that drives BRAF binding to CRAF and paradoxical activation of MEK/ERK. This key experiment will be replicated in Protocol 4.

Packer and colleagues extended the work of Heidorn and colleagues to examine if other more broadly targeted tyrosine kinase inhibitors were also able to paradoxically activate the RAS-RAF pathway. They observed paradoxical pathway activation in D04 cells after treatment with imatinib, nilotinib, dasatinib, and the BRAF inhibitor SB590885. As in Heidorn et al., paradoxical activation only occurred in cells with BRAF^WT^ and required active RAS, as knockdown of NRAS abrogated the effect. Interestingly, while Heidorn and colleagues reported that knockdown of CRAF alone was able to block paradoxical activation, Packer and colleagues reported that only combined knockdown of BRAF and CRAF was able to block paradoxical activation (Packer et al., 2011). Work by Rebocho and colleagues and by Kaplan and colleagues aligned with Heidorn’s findings that silencing of CRAF alone was able to abrogate paradoxical activation (Aplin et al., 2011; Rebocho and Marais 2012). Packer and colleagues also reported that BRAF/CRAF heterodimerization was dependent upon RAS by demonstrating that CRAF^R89L ^was unable to form heterodimers with BRAF (Packer et al., 2011).

Activation of NRAS signaling appears to be a key step in acquired drug resistance, supporting the hypothesis that paradoxical activation can only occur in the context of active RAS signaling. Su and colleagues derived a drug-resistant BRAF^V600E^ melanoma cell line by growing A375 cells in the presence of vemurafenib (PLX4032, a BRAF^V600E^ inhibitor). Interestingly, drug resistance was dependent on expression of CRAF, and the resistant lines that emerged had acquired an activating mutation in KRAS (Su et al., 2012). Nazarian and colleagues also observed the acquisition of activating mutations in NRAS when they derived PLX4032-resistant cell lines (Nazarian et al., 2010). Lidsky and colleagues also showed that increased levels of NRAS were key to vemurafenib resistance, although they did not observe any activating mutations in their resistant cell lines (Lidsky et al., 2014).

## Materials and methods

Unless otherwise noted, all protocol information was derived from the original paper, references from the original paper, or information obtained directly from the authors. An asterisk (*) indicates data or information provided by the Reproducibility Project: Cancer Biology core team. A hashtag (#) indicates information provided by the replicating lab.

### Protocol 1: Treatment of BRAF mutant cells with various RAF inhibitors and assessment of activation of ERK

This protocol describes how to treat NRAS mutant D04 cells and NRAS wild-type cells also carrying the BRAF^V600E^ mutation with various BRAF inhibitors and assess ERK phosphorylation by Western blot, as reported in Figure 1A.

#### Sampling

- The experiment will be performed independently at least three times for a final power of at least 80%. The original data is qualitative, thus to determine an appropriate number of replicates to initially perform, sample sizes based on a range of potential variance was determined.
  - See Power calculations for details.
- Each experiment consists of eight cohorts:
  - Cohort 1: D04 cells treated with DMSO
  - Cohort 2: D04 cells treated with PD184352
  - Cohort 3: D04 cells treated with sorafenib
  - Cohort 4: D04 cells treated with SB590885
  - Cohort 5: A375 cells treated with DMSO
  - Cohort 6: A375 cells treated with PD184352
  - Cohort 7: A375 cells treated with sorafenib
  - Cohort 8: A375 cells treated with SB590885
- Each cohort will be probed for ppERK and ERK2 by Western blot.

#### Materials and reagents

**Table:** 

Reagent	Type	Manufacturer	Catalog #	Comments	
D04 cells	Cells	Provided by Chris Schmidt, Queensland Institute of Medical Research (QIMR) Berghofer, Australia			
A375 cells	Cells	ATCC			
RPMI	Cell culture media	Life Technologies	21875-034		
DMEM	Cell culture media	Life Technologies	41966-029		
FBS	Reagent	Life Technologies	10270106		
35-mm culture plates	Material	Corning	CLS430165	Original not specified	
Sorafenib	Drug	Selleckchem	S7397		
PD184352	Drug	Selleckchem	S1020		
SB590885	Drug	Selleckchem	S2220	*Replaces Plexxion 885-A	
DMSO	Reagent	Fisher Scientific	D128-500	Original not specified	
PBS	Reagent	Gibco	10010-023	Original not specified	
Tris-HCl	Chemical	Specific brand information will be left up to the discretion of the replicating lab and recorded later			
NaCl	Chemical				
Igepal	Chemical				
Na_3_VO_4_	Chemical				
NaF	Chemical				
Leupeptin	Chemical				
Bradford Assay	Detection Assay	Bio-Rad Laboratories	5000001		Original not specified
NuPAGE Sample buffer	Buffer	Invitrogen	NP0007		Original not specified
SDS-Page gel (4–12%)	Western blot reagent	Invitrogen	NP0322BOX		Original not specified
Nitrocellulose membrane (iBlot)	Western blot reagent	Invitrogen	IB301002		Original not specified
Ponceau stain	Western blot reagent	Sigma-Aldrich	P7170-1L		Original not specified
Tris	Chemical	Sigma-Aldrich	T6066		Original not specified
Tween-20	Chemical	Sigma-Aldrich	P1379		Original not specified
Mouse α-ppERK1/2	Antibody	Cell Signaling Technology	9106		Replaces Sigma M8159
Rabbit α-ERK1/2	Antibody	Cell Signaling Technology	9102		Replaces Santa Cruz Bio sc-154
HRP-conjugated secondary antibody	Western blot reagent	Bio-Rad	170-5047		Original not specified
ECL Detection Kit	Western blot reagent	Invitrogen	32132		Original not specified
					

*Suggested as suitable replacement by original authors by personal communication.

#### Procedure

- All cells will be sent for mycoplasma testing and STR profiling.
- D04 cells are maintained in RPMI supplemented with 10% FBS.
- A375 cells are maintained in DMEM supplemented with 10% FBS.
  - All cell lines are maintained at 37°C with 10% CO_2_.
- Sorafenib, PD184352, and SB590885 are dissolved in DMSO.

1. Seed 1.0-2 x 10^5^ cells per well of a six-well tissue culture plate (cells should be 80% confluent at the time of drug treatment).
2. Treat cells with drug or equivalent volume vehicle (DMSO, <0.2%) for 4.
  1. 10 µM Sorafenib
  2. 1 µM SB590885
  3. 1 µM PD184352
3. Lyse cells
  1. Place cells on ice and aspirate media.
  2. Wash three times with ice-cold PBS.
  3. Scrape cells into 50–200 µl of Nonidet P40 extraction buffer.
    1. NP40 extraction buffer: 50 mM Tris-HCl, pH 7.5, 150 mM NaCl, 0.55 (v/v) Igepal, 5 mM NaF, 0.2 mM Na_3_VO_4_, 5 µg/ml leupeptin
    2. Incubate on ice for 5min.
  4. Shear cells by passing through a pipette tip several times.
  5. Centrifuge samples at 20,000 x *g* for 5min at 4°C.
  6. Harvest the soluble fraction for further analysis.
  7. ^#^Quantify protein concentration using a Bradford assay.
4. Analyze cell lysates by Western blot for phospho-ERK and total ERK.
  1. Load equal amounts of all samples (30–50 µg; approximately half of the lysate) mixed with 4x sample buffer and boiled at 90°C for 5–10min on a ^#^4–12% SDS-Page gel.
    1. ^#^Run at ^#^140v for 55min.
  2. ^#^Transfer to a nitrocellulose membrane at 250 mA for 1 hr
    *Confirm protein transfer by Ponceau staining and image membrane.
  3. ^#^Block membrane in 5% non-fat dried milk in TBST (20 mM Tris pH 7.5, 136 mM NaCl, 0.1% Tween-20).
  4. Incubate membrane at 4°C overnight with antibodies against:
    1. Mouse α-ppERK1/2: 1:1000 dilution
    2. ^#^Rabbit α-ERK1/2: 1:1000 dilution
5. ^#^Incubate with HRP-conjugated secondary antibody diluted 1:10,000 in 1X TBS for 1 hr at room temperature.
  1. Rinse the membrane twice with TBST.
  2. Wash the membrane twice with TBST for 5 min each.
6. ^#^Visualize bands with ECL detection kit according to manufacturer’s protocol.
  1. Quantify band intensity.
  2. Normalize pERK to ERK 1/2 for each condition.
7. Repeat independently two additional times.

#### Deliverables

- Data to be collected:
  - Protein quantification results from Bradford assay.
  - Images of Ponceau stained membranes.
  - Raw images of whole gels with ladders included (as reported in Figure 1A).
  - Densitometric quantification of all bands.

#### Confirmatory analysis plan

- Statistical Analysis of the Replication Data:

Note: At the time of analysis, we will perform the Shapiro-Wilk test and generate a quantile-quantile plot to assess the normality of the data. We will also perform Levene’s test to assess homoscedasticity. If the data appears skewed, we will perform a transformation in order to proceed with the proposed statistical analysis. If this is not possible, we will perform the equivalent non-parametric test listed.

- Two-way ANOVA on normalized pERK values (to ERK1/2) in A375 or D04 cells treated with PD184352, sorafenib, SB590885, or vehicle (DMSO) with the following planned contrasts with the Bonferroni correction:
  - Normalized pERK band intensity in A375 cells:
    - Vehicle treatment vs. all three drug treatments (PD184352, sorafenib, and SB590885)
  - Normalized pERK band intensity in D04 cells:
    - Vehicle treatment vs. PD184352 and SB590885 treatments
    - Vehicle treatment vs. sorafenib treatment
- Meta-analysis of original and replication attempt effect sizes:
  - The replication data (mean and 95% confidence interval) will be plotted with the original quantified data value displayed as a single point on the same plot for comparison.

#### Known differences from the original study

The replication attempt will use D04 and A375 cells and will exclude MM415, MM485, and WM852 cells. It will also exclude the drug PLX4720 and will replace 885-A with its analogue SB590885. The original authors suggest they have found similar results with this analogue (personal communication with Dr. Dhomen). All known differences, if any, are listed in the 'Materials and reagents' section above with the originally used item listed in the comments section. The comments section also lists if the source of original item was not specified. All differences have the same capabilities as the original and are not expected to alter the experimental design.

#### Provisions for quality control

All data obtained from the experiment - raw data, data analysis, control data, and quality control data - will be made publicly available, either in the published manuscript or as an open access dataset available on the Open Science Framework (https://osf.io/b1aw6/). Cells will be sent for mycoplasma testing confirming lack of contamination and STR profiling confirming cell line authenticity. The transfer efficiency during the Western blot procedure will be monitored by Ponceau staining.

### Protocol 2: Treatment of NRAS or CRAF silenced D04 cells with SB590885 and assessment of MEK and ERK phosphorylation

This protocol describes treatment of D04 cells transfected with siRNAs targeting NRAS or CRAF with SB590885 and assessment of those cells for activation of MEK and ERK by Western blot, as reported in Figure 1B.

#### Sampling

- The experiment will be performed independently at least four times for a final power of at least 80%. The original data is qualitative, thus to determine an appropriate number of replicates to initially perform, sample sizes based on a range of potential variance was determined.
  - See Power calculations for details.
- Each experiment consists of six cohorts:
  - Cohort 1: control silenced D04 cells
  - Cohort 2: control silenced D04 cells treated with SB590885
  - Cohort 3: NRAS silenced D04 cells
  - Cohort 4: NRAS silenced D04 cells treated with SB590885
  - Cohort 5: CRAF silenced D04 cells
  - Cohort 6: CRAF silenced D04 cells treated with SB590885
- Each cohort will be probed for NRAS, CRAF, ppMEK, α ppERK, and tubulin by Western blot

#### Materials and reagents

**Table:** 

Reagent	Type	Manufacturer	Cat. No.		Comments
D04 cells	Cells	Provided by Chris Schmidt, Queensland Institute of Medical Research (QIMR) Berghofer, Australia			
RPMI	Cell culture media	Life Technologies	21875-034		
FBS	Reagent	Life Technologies	10270106		
SB590885	Drug	Selleckchem	S2220	*Replaces Plexxion 885-A	
DMSO	Reagent	Fisher Scientific	D128-500		Original not specified
35 mm tissue culture dishes	Materials	Corning	CLS430165		Original not specified
INTERFERin	Reagent	Polyplus Transfection	409-01		
CRAF siRNA	siRNA	Synthesis left to the discretion of the replicating lab and will be recorded later			5’-AAGCACGCTTAGATTG GAATA-3’
NRAS siRNA	siRNA	Synthesis left to the discretion of the replicating lab and will be recorded later			5’-CATGGCACTGTACTCT TCTCG-3’
Scrambled siRNA	siRNA	Synthesis left to the discretion of the replicating lab and will be recorded later			5’-AAACCGTC GATTTCACCCGGG-3’
PBS	Reagent	Gibco	10010-023		Original not specified
Tris-HCl	Chemical	Specific brand information will be left up to the discretion of the replicating lab and recorded later			
NaCl	Chemical				
Igepal	Chemical				
Na_3_VO_4_	Chemical				
NaF	Chemical				
Leupeptin	Chemical				
Bradford Assay	Detection Assay	Bio-Rad Laboratories	5000001		Original not specified
NuPAGE Sample buffer	Buffer	Invitrogen	NP0007		Original not specified
SDS-Page gel (4–12%)	Western blot reagent	Invitrogen	NP0322BOX		Original not specified
Nitrocellulose membrane (iBlot)	Western blot reagent	Invitrogen	IB301002		Original not specified
Ponceau stain	Western blot reagent	Sigma-Aldrich	P7170-1L		Original not specified
Tris	Chemical	Sigma-Aldrich	T6066		Original not specified
Tween-20	Chemical	Sigma-Aldrich	P1379		Original not specified
Mouse α NRAS (C-20)	Antibody	Santa Cruz Biotechnology	sc-159		
Mouse α CRAF	Antibody	BD Transduction Laboratories	610152		
Rabbit α ppMEK1/2	Antibody	Cell Signaling Technology	9121		
Mouse α ppERK1/2	Antibody	Sigma	M8159		
Mouse α tubulin	Antibody	Sigma	T5168		
HPR-conjugated secondary antibody	Western blot reagent	Bio-Rad	170-5047	Original not specified	
ECL Detection Kit	Western blot reagent	Invitrogen	32132	Original not specified	
					

#### Procedure

##### Notes

- All cells will be sent for mycoplasma testing and STR profiling.
- D04 cells are maintained in RPMI supplemented with 10% FBS.
  - All cell lines are maintained at 37°C with 10% CO_2_.
- SB590885 is dissolved in DMSO.

1. Seed 3 x 10^5^ D04 cells per 35-mm plate in 2 ml media.
  1. Let incubate overnight.
2. The next morning, prepare siRNA transfection mixture with INTERFERin according to the manufacturer’s protocol, summarized here:
  1. Mix 0.6 µl of 20 µM siRNA with 6 µl INTERERin and 200 µl of serum-free media in RNAse-free tubes.
    1. CRAF siRNA: 5’-AAGCACGCTTAGATTGGAATA-3’
    2. NRAS siRNA: 5’-CATGGCACTGTACTCTTCTCG-3’
    3. Scrambled siRNA control: 5’-AAACCGTC GATTTCACCCGGG-3’
  2. Vortex mixture for 10 s.
  3. Incubate for 5 to 10 min.
  4. Add mixture dropwise to seeded cells in complete media.
  5. Incubate overnight.
3. The next day after transfection, replace with serum free media.
4. 48 hr after siRNA transfection, treat cells with 1 µM SB590885 or equivalent volume vehicle (DMSO, <0.2%) for 4 hr.
5. Lyse cells and harvest extracts as described in Protocol 1 Step 3.
6. Perform Western blots on cell extracts as described in Protocol 1 Step 4.
  - Blot membranes with the following antibodies:
    1. Rabbit α ppMEK: 1:1000 dilution
    2. Rabbit α ppERK: 1:1000 dilution
    3. Mouse α tubulin: 1:5000 dilution

**Table:** 

	Western blot antibody multiplexing			
	POI		Loading control	
Combination	Description	Working conc.	Description	Working conc.
1	Rabit anti-ppMEK (45 kDa)	1:1000	Mouse anti-tubulin (50 kDa)	1:5000
2	Rabbit anti-ppERK (42, 44 kDa)	1:1000	Mouse anti-tubulin (50 kDa)	1:5000
    4. Strip gels with glycine buffer (pH 3.0) containing 1%SDS
    5. Confirm complete stripping and image membranes, block with milk/TBST, and re-probe each gel with one of the following antibodies:
      1. Mouse α NRAS: 1:250 dilution
      2. Mouse α CRAF: 1:1000 dilution
  - Quantify band intensity.
  - Normalize NRAS, CRAF, ppMEK, and ppERK to tubulin for each condition.
7. Repeat independently three additional times.

#### Deliverables

- Data to be collected:
  - Protein quantification results from Bradford assay.
  - Images of Ponceau stained membranes.
  - Images of whole gels with ladder (as reported in Figure 1B).
  - Densitometric quantification of all bands.

#### Confirmatory analysis plan

- Statistical Analysis of the Replication Data:

Note: At the time of analysis, we will calculate Pearson’s *r* to check for correlation between the dependent variables, a scatter plot to assess linearity, and a Box’s M test to check for equality of covariance matrices. We will also perform the Shapiro-Wilk test and generate a quantile-quantile plot to assess the normality of the data. We will perform Levene’s test to assess homoscedasticity. If the data appears skewed, we will perform a transformation in order to proceed with the proposed statistical analysis. If this is not possible, we will perform the equivalent non-parametric test.

- One-way MANOVA comparing the differences between SB590885 treatment and vehicle treatment of normalized band intensities for pMEK and pERK levels in D04 cells transfected with siRNA for NRAS, CRAF, or control with the following Bonferroni-corrected comparisons:
  - Difference in normalized ppMEK levels between SB590885 and vehicle treatment:
    - Control siRNA compared to NRAS siRNA.
    - Control siRNA compared to CRAF siRNA.
  - Difference in normalized ppERK levels between SB590885 and vehicle treatment:
    - Control siRNA compared to NRAS siRNA.
    - Control siRNA compared to CRAF siRNA
- Meta-analysis of original and replication attempt effect sizes:
  - The replication data (mean and 95% confidence interval) will be plotted with the original quantified data value displayed as a single point on the same plot for comparison.

#### Known differences from the original study

The replication will replace 885-A with its analogue SB590885. The original authors suggest they have found similar results with this analogue (personal communication with Dr. Dhomen). All known differences, if any, are listed in the 'Materials and reagents' section above with the originally used item listed in the comments section. The comments section also lists if the source of original item was not specified. All differences have the same capabilities as the original and are not expected to alter the experimental design.

#### Provisions for quality control

Cells will be sent for mycoplasma testing confirming lack of contamination and STR profiling confirming cell line authenticity. The transfer efficiency during the Western blot procedure will be monitored by Ponceau staining. The membrane will be imaged after stripping to confirm and measure background. All data obtained from the experiment - raw data, data analysis, control data, and quality control data - will be made publicly available, either in the published manuscript or as an open access dataset available on the Open Science Framework (https://osf.io/b1aw6/).

### Protocol 3: Immunoprecipitation of CRAF from SB590885 treated D04 cells expressing myc-tagged CRAF^WT^ or CRAF^R89L^

This protocol describes how to immunoprecipitate myc-tagged CRAF^WT^ or CRAF^R89L^, a mutant form that cannot bind RAS, from D04 cells and probe the pulldown for BRAF, as reported in Figure 3A.

#### Sampling

- The experiment will be performed independently at least three times for a final power of at least 80%. The original data is qualitative, thus to determine an appropriate number of replicates to initially perform, sample sizes based on a range of potential variance was determined.
  - See Power calculations for details.
- Each experiment consists of six cohorts:
  - Cohort 1: D04 cells transfected with myc-tagged CRAF^WT^ treated with SB590885
  - Cohort 2: D04 cells transfected with myc-tagged CRAF^WT^ treated with DMSO
  - Cohort 3: D04 cells transfected with myc-tagged CRAF^R89L^ treated with SB590885
  - Cohort 4: D04 cells transfected with myc-tagged CRAF^R89L^ treated with DMSO
  - Cohort 5: D04 cells transfected with empty vector treated with SB590885
  - Cohort 6: D04 cells transfected with empty vector treated with DMSO
- Each cohort will be immunoprecipitated for myc-tagged CRAF and immunoprecipitate and lysates probed for BRAF and myc.

#### Materials and reagents

**Table:** 

Reagent	Type	Manufacturer	Catalog #	Comments
D04 cells	Cells	Provided by Chris Schmidt, Queensland Institute of Medical Research (QIMR) Berghofer, Australia		
SB590885	Drug	Selleckchem	S2220	*Replaces Plexxion 885-A
DMSO	Reagent	Fisher Scientific	D128-500	Original not specified
RPMI	Media	Life Technologies	21875-034	
FBS	Reagent	Life Technologies	10270106	
Effectene Transfection Reagent	Reagent	Qiagen	301425	Replaces Cell Line Nucleofector Kit V (10 RCT) Lonza VACA1003
35 mm culture dishes	Materials	Corning	CLS430165	Original not specified
Myc-CRAF^WT^ vector	Plasmid	Shared by original authors		
Myc-CRAF^R89L^ vector	Plasmid	Shared by original authors		
Empty vector	Plasmid	Shared by original authors		
PBS	Reagent	Gibco	10010-023	Original not specified
Tris-HCl	Chemical	Specific brand information will be left up to the discretion of the replicating lab and recorded later		
NaCl	Chemical			
Igepal	Chemical			
Na_3_VO_4_	Chemical			
NaF	Chemical			
Leupeptin	Chemical			
Rabbit α myc	Antibody	Abcam	ab9106	
Mouse α BRAF (F-7)	Antibody	Santa Cruz Biotechnology	sc-5284	
Mouse α myc (9B11) (HRP conjugate)	Antibody	Cell Signaling Technology	2040	
Protein G sepharose beads	Materials	Sigma	P3296	
NuPAGE Sample buffer	Buffer	Invitrogen	NP0007	Original not specified
SDS-Page gel (4–12%)	Western blot reagent	Invitrogen	NP0322BOX	Original not specified
Nitrocellulose membrane (iBlot)	Western blot reagent	Invitrogen	IB301002	Original not specified
Ponceau stain	Western blot reagent	Sigma-Aldrich	P7170-1L	Original not specified
Tris	Chemical	Sigma-Aldrich	T6066	Original not specified
Tween-20	Chemical	Sigma-Aldrich	P1379	Original not specified
HPR-conjugated secondary antibody	Western blot reagent	Bio-Rad	170-5047	Original not specified
ECL Detection Kit	Western blot reagent	Invitrogen	32132	Original not specified

#### Procedure

##### Notes

- All cells will be sent for mycoplasma testing and STR profiling.
- D04 cells are maintained in RPMI supplemented with 10% FBS.
  - All cell lines are maintained at 37°C with 10% CO_2_.
- SB590885 is dissolved in DMSO.

1. Transfect D04 cells with vectors containing myc-tagged CRAF^wt^ or CRAF^R89L^.
  1. ^#^Plate 1x10^6^ cells per well of a six-well plate with 1.6 ml media 1 day before transfection. The cells should be 40–80% confluent on the day of transfection.
  2. ^#^On the day of transfection, dilute 0.4 µg of DNA for each vector in TE buffer, pH 7 with the DNA-condensation buffer, Buffer EC, to a total volume of 100 μl. Add 3.2 μl Enhancer and mix by vortexing.
    1. Empty vector
    2. Myc-CRAF^WT^ vector
    3. Myc-CRAF^R89L^ vector
  3. ^#^Incubate at room temperature for 5 min, centrifuge quickly.
  4. ^#^Add 10 µl Effectene Transfection Reagent to the DNA-Enhancer mixture and mix by pipetting.
  5. ^#^Incubate at room temperature for 10 min.
  6. ^#^Gently aspirate the medium from the plated cells and wash once with 2 ml PBS. Add 1.6 ml fresh medium to the cells.
  7. ^#^Add 600 µl medium to the tube containing transfection complexes and mix by pipetting. Immediately add transfection complexes drop-wise onto plated cells. Gently swirl to mix.
  8. ^#^Incubate for 18 hr after transfection. Replace with fresh medium.
2. 48 hr after transfection, treat cells with 1 µM SB590885 or equivalent volume vehicle (DMSO, <0.2%) for 4 hr.
3. Lyse cells and prepare cell lysate as described in Protocol 1 Step 3.
  1. Save 5–15 µg protein from each lysate to confirm transfection by Western blot below.
4. Immunoprecipitate myc-tagged CRAF proteinsNote: 2-3 35 mm wells of protein lysed in 300 µl NP40 buffer are needed for IP reaction.
  1. Immunoprecipitate the Myc-tagged proteins by adding 2 µg rabbit anti-myc antibody and incubate overnight at 4°C.
  2. Capture the antibody-protein complex by adding 20 µl of a 1:1 Protein G sepharose 4B beads mixture in NP40 extraction buffer.
    1. NP40 extraction buffer: 50 mM Tris-HCl, pH 7.5, 150 mM NaCl, 0.55 (v/v) Igepal, 5 mM NaF, 0.2 mM Na_3_VO_4_, 5 µg/ml leupeptin.
    2. Incubate on ice for 5 min.
    3. Mix on a rotating wheel for 2 hr at 4°C.
  3. Wash IPs three times with 300 µl NP40 extraction buffer.
  4. Elute protein complex from beads with NuPage sample buffer
5. Run IPs and lysate on an SDS-PAGE gel as described in Protocol 1 Step 4.
  1. Probe with the following antibodies:
    1. Mouse α BRAF: 1:2000 dilution
    2. Mouse α myc: 1:1000 dilution
  2. Quantify band intensity.
  3. Normalize IP α BRAF to IP α myc-CRAF for each condition from IP band intensities.
6. Repeat independently two additional times.

#### Deliverables

- Data to be collected:
  - Protein quantification results from Bradford assay.
  - Images of Ponceau stained membranes.
  - Transfection QC images of whole gels with ladder.
  - Images of whole gels with ladder (as reported in Figure 3A).
  - Densitometric quantification of all bands.

#### Confirmatory analysis plan

- Statistical Analysis of the Replication Data:

Note: At the time of analysis, we will perform the Shapiro-Wilk test and generate a quantile-quantile plot to assess the normality of the data. We will also perform Levene’s test to assess homoscedasticity. If the data appears skewed, we will perform a transformation in order to proceed with the proposed statistical analysis. If this is not possible, we will perform the equivalent non-parametric test listed.

- Two-way ANOVA comparing normalized IP BRAF (to IP α myc) band intensity in D04 cells transfected with Myc-CRAF^WT^ vector or Myc-CRAF^R89L^ vector with or without SB590885 drug treatment, and the following Bonferroni-corrected comparisons:
  - Normalized IP BRAF band intensity in cells with Myc-CRAF^WT^ vector with SB590885 treatment vs. vehicle treatment.
  - Normalized IP BRAF band intensity in cells with Myc- CRAF^R89L^ vector with SB590885 treatment vs. vehicle treatment.
- Meta-analysis of original and replication attempt effect sizes:
  - The replication data (mean and 95% confidence interval) will be plotted with the original quantified data value displayed as a single point on the same plot for comparison.

#### Known differences from the original study

The transfection method using Nucleofectin Solution V and electroporation will be replaced with a lipid-based method using Effectene Transfection Reagent, and protocol will be changed according to Manufacturer’s instructions. This difference in transfection protocol might lead to differences in expression that could lead to differences in results. The replication will replace 885-A with its analogue SB590885. The original authors suggest they have found similar results with this analogue (personal communication with Dr. Dhomen). All known differences, if any, are listed in the 'Materials and reagents' section above with the originally used item listed in the comments section. The comments section also lists if the source of original item was not specified. All differences have the same capabilities as the original and are not expected to alter the experimental design.

#### Provisions for quality control

Cells will be sent for mycoplasma testing confirming lack of contamination and STR profiling confirming cell line authenticity. Transfection will be confirmed with Western blot. The transfer efficiency during the Western blot procedure will be monitored by Ponceau staining. All data obtained from the experiment - raw data, data analysis, control data, and quality control data - will be made publicly available, either as a published manuscript or as an open access dataset available on the Open Science Framework (https://osf.io/b1aw6/).

### Protocol 4: Immunoprecipitation of BRAF from SB590885 treated D04 cells expressing myc-tagged BRAF^WT^ or BRAF^R188L^

This protocol describes how to immunoprecipitate myc-tagged BRAF^WT^ or BRAF^R188L^, a mutant form that cannot bind RAS, from D04 cells and probe the pulldown for CRAF, as reported in Figure 3B.

#### Sampling

- The experiment will be performed independently at least three times for a final power of at least 80%. The original data is qualitative, thus to determine an appropriate number of replicates to initially perform, sample sizes based on a range of potential variance was determined.
  - See Power calculations for details.
- Each experiment consists of six cohorts:
  - Cohort 1: D04 cells transfected with myc-tagged BRAF^WT^ treated with SB590885
  - Cohort 2: D04 cells transfected with myc-tagged BRAF^WT^ treated with DMSO
  - Cohort 3: D04 cells transfected with myc-tagged BRAF^R188L^ treated with SB590885
  - Cohort 4: D04 cells transfected with myc-tagged BRAF^R188L^ treated with DMSO
  - Cohort 5: D04 cells transfected with empty vector treated with SB590885
  - Cohort 6: D04 cells transfected with empty vector treated with DMSO
- Each cohort will be immunoprecipitated for myc-tagged BRAF and immunoprecipitate and lysates probed for CRAF and myc.

#### Materials and reagents

**Table:** 

Reagent	Type	Manufacturer	Catalog #	Comments
D04 cells	Cells	Provided by Chris Schmidt, Queensland Institute of Medical Research (QIMR) Berghofer, Australia		
SB590885	Drug	Selleckchem	S2220	*Replaces Plexxion 885-A
DMSO	Reagent	Fisher Scientific	D128-500	Original not specified
RPMI	Media	Life Technologies	21875-034	
FBS	Reagent	Life Technologies	10270106	
Effectene Transfection Reagent	Reagent	Qiagen	301425	Replaces Cell Line Nucleofector Kit V (10 RCT) Lonza VACA1003
35-mm culture dishes	Materials	Corning	CLS430165	Original not specified
Myc-BRAF^WT^ vector	Plasmid	Shared by original authors		
Myc-BRAF^R188L^ vector	Plasmid	Shared by original authors		
Empty vector	Plasmid	Shared by original authors		
PBS	Reagent	Gibco	10010-023	Original not specified
Tris-HCl	Chemical	Specific brand information will be left up to the discretion of the replicating lab and recorded later		
NaCl	Chemical			
Igepal	Chemical			
Na_3_VO_4_	Chemical			
NaF	Chemical			
Leupeptin	Chemical			
Rabbit anti-myc	Antibody	Abcam	ab9106	
mouse anti-CRAF	Antibody	BD Transduction Laboratories	610152	
Mouse α myc (9B11) (HRP conjugate)	Antibody	Cell Signaling Technology	2040	
Protein G sepharose beads	Materials	Sigma	P3296	
NuPAGE Sample buffer	Buffer	Invitrogen	NP0007	Original not specified
SDS-Page gel (4–12%)	Western blot reagent	Invitrogen	NP0322BOX	Original not specified
Nitrocellulose membrane (iBlot)	Western blot reagent	Invitrogen	IB301002	Original not specified
Ponceau stain	Western blot reagent	Sigma-Aldrich	P7170-1L	Original not specified
Tris	Chemical	Sigma-Aldrich	T6066	Original not specified
Tween-20	Chemical	Sigma-Aldrich	P1379	Original not specified
HPR-conjugated secondary antibody	Western blot reagent	Bio-Rad	170-5047	Original not specified
ECL Detection Kit	Western blot reagent	Invitrogen	32132	Original not specified

#### Procedure

Notes:

- All cells will be sent for mycoplasma testing and STR profiling.
- D04 cells are maintained in RPMI supplemented with 10% FBS.
  - All cell lines are maintained at 37°C with 10% CO_2_.
- SB590885 is dissolved in DMSO.

1. Transfect D04 cells with vectors containing myc-tagged BRAF^wt^ or BRAF^R188L^ as described in Protocol 3 Step 1.
2. 48 hr after transfection, treat cells with 1 µM SB590885 or equivalent volume vehicle (DMSO, <0.2%) for 4 hr.
3. Lyse cells and prepare cell lysate as described in Protocol 1 Step 3.
  1. Save 5-15 μg protein from each lysate to confirm transfection by Western blot below.
4. Immunoprecipitate myc-tagged CRAF proteinsNote: 2-3 35 mm wells of protein lysed in 300 µl NP40 buffer are needed for IP reaction.
  1. Immunoprecipitate the Myc-tagged proteins by adding 2 µg rabbit anti-myc antibody and incubate overnight at 4°C.
  2. Capture the antibody-protein complex by adding 20 µl of a 1:1 Protein G sepharose 4B beads mixture in NP40 extraction buffer.
    1. NP40 extraction buffer: 50 mM Tris-HCl, pH 7.5, 150 mM NaCl, 0.55 (v/v) Igepal, 5 mM NaF, 0.2 mM Na_3_VO_4_, 5 µg/ml leupeptin.
    2. Incubate on ice for 5 min.
    3. Mix on a rotating wheel for 2 hr at 4°C.
  3. Wash IPs three times with 300 µl NP40 extraction buffer.
  4. Elute protein complex from beads with NuPage sample buffer
5. Run IPs and lysate on an SDS-PAGE gel as described in Protocol 1 Step 4.
  1. Probe with the following antibodies:
    1. Mouse α CRAF: 1:1000 dilution
    2. Mouse α myc: 1:1000 dilution
  2. Quantify band intensity.
  3. Normalize IP α CRAF to IP α myc-BRAF for each condition from IP band intensities.
6. Repeat independently two additional times.

#### Deliverables

- Data to be collected:
  - Protein quantification results from Bradford assay.
  - Images of Ponceau stained membranes.
  - Transfection QC images of whole gels with ladder.
  - Images of whole gels with ladder (as reported in Figure 3A).
  - Densitometric quantification of all bands.

#### Confirmatory analysis plan

- Statistical Analysis of the Replication Data:

Note: At the time of analysis, we will perform the Shapiro-Wilk test and generate a quantile-quantile plot to assess the normality of the data. We will also perform Levene’s test to assess homoscedasticity. If the data appears skewed, we will perform a transformation in order to proceed with the proposed statistical analysis. If this is not possible, we will perform the equivalent non-parametric test listed.

- Two-way ANOVA comparing normalized IP CRAF (to IP α myc) band intensity in D04 cells transfected with Myc-BRAF^WT^ vector or Myc-BRAF^R188L^ vector with or without SB590885 drug treatment, and the following Bonferroni-corrected comparisons:
  - Normalized IP CRAF band intensity in cells with Myc-BRAF^WT^ vector with SB590885 treatment vs. vehicle treatment.
  - Normalized IP CRAF band intensity in cells with Myc- BRAF^R188L^ vector with SB590885 treatment vs. vehicle treatment.
- Meta-analysis of original and replication attempt effect sizes:
  - The replication data (mean and 95% confidence interval) will be plotted with the original quantified data value displayed as a single point on the same plot for comparison.

#### Known differences from the original study

The transfection method using Nucleofectin Solution V and electroporation will be replaced with a lipid-based method using Effectene Transfection Reagent, and protocol will be changed according to Manufacturer’s instructions. The replication will replace 885-A with its analogue SB590885. The original authors suggest they have found similar results with this analogue (personal communication with Dr. Dhomen). All known differences, if any, are listed in the 'Materials and reagents' section above with the originally used item listed in the comments section. The comments section also lists if the source of original item was not specified. All differences have the same capabilities as the original and are not expected to alter the experimental design.

#### Provisions for quality control

Cells will be sent for mycoplasma testing confirming lack of contamination and STR profiling confirming cell line authenticity. Transfection will be confirmed with Western blot. The transfer efficiency during the Western blot procedure will be monitored by Ponceau staining. All data obtained from the experiment - raw data, data analysis, control data, and quality control data - will be made publicly available, either as a published manuscript or as an open access dataset available on the Open Science Framework (https://osf.io/b1aw6/).

### Protocol 5: Expression of BRAF kinase dead mutant in D04 cells and its effect on CRAF binding

This protocol describes how to transiently express myc-tagged BRAF^WT^ or BRAF^D59A^ in D04 cells and assess CRAF binding by immunoprecipitation and blotting, as reported in Figure 4D.

#### Sampling

- The experiment will be performed independently at least three times for a minimum power of 80%. The original data is qualitative, thus to determine an appropriate number of replicates to initially perform, sample sizes based on a range of potential variance was determined.
  - See Power Calculations for details.
- Each experiment consists of three cohorts:
  - Cohort 1: D04 cells transfected with myc-tagged BRAF^WT^
  - Cohort 2: D04 cells transfected with myc-tagged BRAF^D594A^
  - Cohort 3: D04 cells transfected with empty vector
  - Untreated cells are immunoprecipitated with α myc and levels of myc-BRAF and CRAF are assessed by immunoblotting.

#### Materials and reagents

**Table:** 

Reagent	Type	Manufacturer	Catalog #	Comments
D04 cells	Cells	Provided by Chris Schmidt, Queensland Institute of Medical Research (QIMR) Berghofer, Australia		
RPMI	Media	Life Technologies	21875-034	
FBS	Reagent	Life Technologies	10270106	
Effectene Transfection Reagent	Reagent	Qiagen	301425	Replaces Cell Line Nucleofector Kit V (10 RCT) Lonza VACA1003
Myc-BRAF^WT^ vector	Plasmid	Shared by original author		
Myc-BRAF^D594A^ vector	Plasmid	Shared by original author		
Empty vector	Plasmid	Shared by original author		
35 mm culture dishes	Materials			
PBS	Reagent	Gibco	10010-023	Original not specified
Tris-HCl	Chemical	Specific brand information will be left up to the discretion of the replicating lab and recorded later		
NaCl	Chemical			
Igepal	Chemical			
Na_3_VO_4_	Chemical			
NaF	Chemical			
Leupeptin	Chemical			
Rabbit α myc	Antibody	Abcam	ab9106	
Mouse α CRAF (for Western blotting)	Antibody	BD Transduction Laboratories	610152	
Mouse α myc (9B11) (HRP conjugate)	Antibody	Cell Signaling Technology	2040	
Protein G sepharose beads	Materials	Sigma	P3296	
NuPAGE Sample buffer	Buffer	Invitrogen	NP0007	Original not specified
SDS-Page gel (4–12%)	Western blot reagent	Invitrogen	NP0322BOX	Original not specified
Nitrocellulose membrane (iBlot)	Western blot reagent	Invitrogen	IB301002	Original not specified
Ponceau stain	Western blot reagent	Sigma-Aldrich	P7170-1L	Original not specified
Tris	Chemical	Sigma-Aldrich	T6066	Original not specified
Tween-20	Chemical	Sigma-Aldrich	P1379	Original not specified
HPR-conjugated secondary antibody	Western blot reagent	Bio-Rad	170-5047	Original not specified
ECL Detection Kit	Western blot reagent	Invitrogen	32132	Original not specified

#### Procedure

Notes:

- All cells will be sent for mycoplasma testing and STR profiling.
- D04 cells are maintained in RPMI supplemented with 10% FBS.
  - All cell lines are maintained at 37°C with 10% CO_2_.

1. Transiently transfect D04 cells with the following vectors as described in Protocol 3 step 1.
  1. Myc-BRAF^WT^ vector
  2. Myc-BRAF^D594A^ vector
  3. Empty vector
2. Lyse cells and prepare cell lysates as described in Protocol 1 Step 3.
  1. Save 5–15 μg protein from each lysate to confirm transfection by Western blot below.
3. Immunoprecipitate myc-tagged BRAF proteins as described in Protocol 3 Step 4.
4. Run IPs and lysate on SDS-PAGE gel as described in Protocol 1 Step 4.
  1. Probe with the following antibodies:
    1. Mouse α CRAF: 1:1000 dilution
    2. Mouse α myc: 1:1000 dilution
  2. Quantify band intensity.
  3. Normalize IP α CRAF to IP α myc-BRAF for each condition from IP band intensities.
5. Repeat independently two additional times.

#### Deliverables

- Data to be collected:
  - Protein quantification results from Bradford assay.
  - Images of Ponceau stained membranes.
  - Images of whole gels (as reported in Figure 4D).
  - Densitometric quantification of all bands.
  - Any data pertaining to cell growth conditions optimization, if performed.

#### Confirmatory analysis plan

- Statistical Analysis of the Replication Data:
  - A two sample Welch’s *t*-test comparing normalized IP CRAF (using IP myc-BRAF band intensity) in D04 cells transfected with Myc-BRAF^WT^ vector vs. Myc-BRAF^D594A^ vector
- Meta-analysis of original and replication attempt effect sizes:
  - The replication data (mean and 95% confidence interval) will be plotted with the original quantified data value displayed as a single point on the same plot for comparison.

#### Known differences from the original study

All known differences, if any, are listed in the 'Materials and reagents' section above with the originally used item listed in the comments section. The comments section also lists if the source of original item was not specified. All differences have the same capabilities as the original and are not expected to alter the experimental design.

#### Provisions for quality control

Cells will be sent for mycoplasma testing confirming lack of contamination and STR profiling confirming cell line authenticity. Transfection will be confirmed with Western blot. The transfer efficiency during the Western blot procedure will be monitored by Ponceau staining. All data obtained from the experiment - raw data, data analysis, control data, and quality control data - will be made publicly available, either as a published manuscript or as an open access dataset available on the Open Science Framework (https://osf.io/b1aw6/). Cells will be sent for mycoplasma testing confirming lack of contamination and STR profiling confirming cell line authenticity.

#### Power calculations

For additional details on power calculations, please see analysis scripts and associated files on the Open Science Framework:

https://osf.io/eaktg/

### Protocol 1

#### Summary of original data

- The original data presented is qualitative (images of Western blots). We used Image Studio Lite (LICOR) to perform densitometric analysis of the presented bands. We then performed a priori power calculations with a range of assumed standard deviations to determine the number of replicates to perform.
- Note: band intensity quantified from the image reported in Figure 1A:

**Table:** 

Cell type	Drug	Band intensity normalized total ERK	Assumed N	
A375	Control	1.3864	3	
	PD	0.0127	3	
	SF	0.0257	3	
	885-A	0.0510	3	
D04	Control	0.1315	3	
	PD	0.0198	3	
	SF	0.0123	3	
	885-A	0.6650	3

- The original data does not indicate the error associated with multiple biological replicates. To identify a suitable sample size, power calculations were performed using different levels of relative variance.

#### Test family

- Two-way ANOVA (2 x 4) fixed effects, special, main effects and interactions; alpha error = 0.05 followed by Bonferroni corrected comparisons

#### Power calculations

- Power calculations were performed using R software version 3.2.1 (R Core Team, 2014) and G*Power (version 3.1.7) (Faul et al., 2007)

**Table:** 

Groups	Estimated variance	F test statistic F_(3,16)_interaction	Partial η^2^	Effect size *f*	A priori power	Total sample size (8 groups)
A375 or D04 cells treated with drugs or control	2%	7743.50	0.9993	38.112	99.9%	9
	15%	137.662	0.9627	5.080	98.8%	10
	28%	39.507	0.8811	2.722	96.0%	11
	40%	19.359	0.7840	1.905	91.6%	12

#### Test family

- *F* test, ANOVA: Fixed effects, special, main effects and interactions, Bonferroni’s correction: alpha error = 0.01667

Power Calculations performed with G*Power software, version 3.1.7 (Faul et al., 2007).

ANOVA F test statistic and partial η^2^performed with R software, version 3.2.1 (Team, 2015). Partial η^2^ calculated from (Lakens, 2013).

For A375 cells, comparisons are between DMSO and all Drug Treatments (PD184352, sorafenib, and 885-A)

**Table:** 

Groups	Cell line	Variance estimate	F test statistic Fc_(1,16)_	Partial η^2^	Effect size *f*	A priori power	Total sample size (8 groups)
DMSO vs all Drug Treatments	A375	2%	34711.2	0.9995	46.58	99.9%	9
	A375	15%	617.09	0.9747	6.210	99.8%	10
	A375	28%	177.10	0.9171	3.327	84.2%	10
	A375	40%	86.78	0.8443	2.329	92.7%	11

For D04 cells, comparisons are between DMSO and PD184352 and sorafenib, and between DMSO and 885-A

**Table:** 

Groups	Cell line	Variance estimate	F test statistic Fc_(1,16)_	Partial η^2^	Effect size *f*	A priori power	Total sample size (8 groups)
DMSO vs. PD184352 and sorafenib	D04	2%	223.55	0.9332	3.7379	90.2%	10
	D04	15%	3.9741	0.1990	0.4984	80.4%	46
	D04	28%	1.1405	0.0665	0.2670	80.0%	150
	D04	40%	0.5589	0.0337	0.1869	80.0%	303
DMSO vs. 885A	D04	2%	3580.31	0.9955	14.959	99.9%	10
	D04	15%	63.6498	0.7991	1.9945	84.0%	11
	D04	28%	18.2668	0.5331	1.0685	80.8%	15
	D04	40%	8.9507	0.3587	0.7479	80.1%	23

- Based on these power calculations, we will run the experiment three times. Each time, we will quantify band intensity. We will determine the standard deviation of band intensity across the biological replicates and combine this with the effect size from the original study to calculate the number of replicates necessary to reach a power of at least 80%. We will then perform additional replicates, if required, to ensure the experiment has more than 80% power to detect the original effect.

### Protocol 2

#### Summary of original data

- The original data presented is qualitative (images of Western blots). We used Image Studio Lite (LICOR) to perform densitometric analysis of the presented bands. We then performed a priori power calculations with a range of assumed standard deviations to determine the number of replicates to perform.
- Note: band intensity quantified from the image reported in Figure 1B:

**Table:** 

Target	siRNA	Band intensity normalized to tubulin for transfected cells treated with 885-A minus DMSO	Assumed N
pMEK	Control	0.836493931	3
	NRAS	0.0695447	3
	CRAF	0.3538748	3
pERK	Control	0.8769868	3
	NRAS	0.498252598	3
	CRAF	0.653649416	3

### Test family

- Due to the lack of raw original data, we are unable to perform power calculations using a MANOVA. We are determining sample size using two one-way ANOVAs.
- Two, one-way ANOVAs (Bonferroni corrected) on the difference in the normalized band intensity for pMEK and pERK separately in transfected cells treated with 885-A minus DMSO followed by Bonferroni corrected comparisons for the following groups:
  - pMEK and pERK each:
    - Compare the difference in band intensity in cells transfected with control siRNA and treated with 885-A minus control siRNA with DMSO (Control siRNA Difference) vs. the difference in band intensity in cells transfected with NRAS siRNA and treated with 885-A minus NRAS siRNA with DMSO (NRAS siRNA Difference)
    - Compare the difference in band intensity in cells transfected with control siRNA and treated with 885-A minus control siRNA with DMSO (Control siRNA Difference) vs. the difference in band intensity in cells transfected with CRAF siRNA and treated with 885-A minus CRAF siRNA with DMSO (CRAF siRNA Difference)

### Power calculations

- Power calculations were performed using R software version 3.1.2 (R Core Team, 2014) and G*Power (version 3.1.7) (Faul et al., 2007)

#### pMEK

- 2% variance:
  - ANOVA: Fixed effects, omnibus, one-way, Bonferroni corrected alpha error = 0.025

**Table:** 

Groups	F test statistic	Partial η^2^	Effect size *f*	A priori power	Total sample size
D04 cells silenced for NRAS or CRAF and exposed to Drug Treatment	F_(2,6)_ = 1019.1	0.9971	18.5426	>99.9%	6 (3 groups)

- Bonferroni- corrected planned comparisons; alpha error = 0.0125

**Table:** 

Group 1	Group 2	Effect size *d*	A priori power	Sample size per group
Control siRNA Difference	NRAS siRNA Difference	36.4575	99.3%^1^	2^1^
Control siRNA Difference	CRAF siRNA Difference	8.6916	99.9%	3

- 15% variance:
  - ANOVA: Fixed effects, omnibus, one-way, Bonferroni corrected alpha error = 0.025

**Table:** 

Groups	F test statistic	Partial η^2^	Effect size *f*	A priori power	Total sample size
D04 cells silenced for NRAS or CRAF and exposed to Drug Treatment	F_(2,6)_ = 72.467	0.9602	4.9118	99.5%	6 (3 groups)

- Bonferroni- corrected planned comparisons; alpha error = 0.0125

**Table:** 

Group 1	Group 2	Effect size *d*	A priori power	Sample size per group
Control siRNA Difference	NRAS siRNA Difference	9.7218	>99.9%	3
Control siRNA Difference	CRAF siRNA Difference	2.3177	80.9%	6

- 28% variance:
  - ANOVA: Fixed effects, omnibus, one-way, Bonferroni corrected alpha error = 0.025

**Table:** 

Groups	F test statistic	Partial η^2^	Effect size *f*	A priori power	Total sample size
D04 cells silenced for NRAS or CRAF and exposed to Drug Treatment	F_(2,6)_ = 20.797	0.8739	2.6325	99.8%	9 (3 groups)

- Bonferroni- corrected planned comparisons; alpha error = 0.0125

**Table:** 

Group 1	Group 2	Effect size *d*	A priori power	Sample size per group
Control siRNA Difference	NRAS siRNA Difference	5.2081	89.9%	3
Control siRNA Difference	CRAF siRNA Difference	1.2416	82.7%	17

- 40% variance:
  - ANOVA: Fixed effects, omnibus, one-way, Bonferroni corrected alpha error = 0.025

**Table:** 

Groups	F test statistic	Partial η^2^	Effect size *f*	A priori power	Total sample size
D04 cells silenced for NRAS or CRAF and exposed to Drug Treatment	F_(2,6)_ = 10.191	0.7726	1.8432	90.8%	9 (3 groups)

- Bonferroni- corrected planned comparisons; alpha error = 0.0125

**Table:** 

Group 1	Group 2	Effect size *d*	A priori power	Sample size per group
Control siRNA Difference	NRAS siRNA Difference	3.6457	89.7%	4
Control siRNA Difference	CRAF siRNA Difference	0.8692	81.4%	32

#### pERK

- 2% variance:
  - ANOVA: Fixed effects, omnibus, one-way, Bonferroni corrected alpha error = 0.025

**Table:** 

Groups	F test statistic	Partial η^2^	Effect size *f*	A priori power	Total sample size
D04 cells silenced for NRAS or CRAF and exposed to Drug Treatment	F_(2,6)_ = 141.13	0.9792	6.8613	>99.9%	6 (3 groups)

- Bonferroni- corrected planned comparisons; alpha error = 0.0125

**Table:** 

Group 1	Group 2	Effect size *d*	A priori power	Sample size per group
Control siRNA Difference	NRAS siRNA Difference	13.6467	90.2%	2
Control siRNA Difference	CRAF siRNA Difference	8.0474	99.9%	3

- 15% variance:
  - ANOVA: Fixed effects, omnibus, one-way, Bonferroni corrected alpha error = 0.025

**Table:** 

Groups	F test statistic	Partial η^2^	Effect size *f*	A priori power	Total sample size
D04 cells silenced for NRAS or CRAF and exposed to Drug Treatment	F_(2,6)_ = 10.036	0.7699	1.8292	90.3%	9 (3 groups)

- Bonferroni- corrected planned comparisons; alpha error = 0.0125

**Table:** 

Group 1	Group 2	Effect size *d*	A priori power	Sample size per group
Control siRNA Difference	NRAS siRNA Difference	3.6391	89.3%	4
Control siRNA Difference	CRAF siRNA Difference	2.1460	83.7%	7

- 28% variance:
  - ANOVA: Fixed effects, omnibus, one-way, Bonferroni corrected alpha error = 0.025

**Table:** 

Groups	F test statistic	Partial η^2^	Effect size *f*	A priori power	Total sample size
D04 cells silenced for NRAS or CRAF and exposed to Drug Treatment	F_(2,6)_ = 2.8802	0.4898	0.9798	86.4%	18 (3 groups)

- Bonferroni- corrected planned comparisons; alpha error = 0.0125

**Table:** 

Group 1	Group 2	Effect size *d*	A priori power	Sample size per group
Control siRNA Difference	NRAS siRNA Difference	1.9495	83.1%	8
Control siRNA Difference	CRAF siRNA Difference	1.1496	81.4%	19

- 40% variance:
  - ANOVA: Fixed effects, omnibus, one-way, Bonferroni corrected alpha error = 0.025

**Table:** 

Groups	F test statistic	Partial η^2^	Effect size *f*	A priori power	Total sample size
D04 cells silenced for NRAS or CRAF and exposed to Drug Treatment	F_(2,6)_ = 1.4113	0.3199	0.6858	82.9%	30 (3 groups)

- Bonferroni- corrected planned comparisons; alpha error = 0.0125

**Table:** 

Group 1	Group 2	Effect size *d*	A priori power	Sample size per group
Control siRNA Difference	NRAS siRNA Difference	1.3647	81.4%	14
Control siRNA Difference	CRAF siRNA Difference	0.8047	81.3%	37

- Based on these power calculations, we will run the experiment four times. Each time, we will quantify band intensity. We will determine the standard deviation of band intensity across the biological replicates and combine this with the effect size from the original study to calculate the number of replicates necessary to reach a power of at least 80%. We will then perform additional replicates, if required, to ensure the experiment has more than 80% power to detect the original effect.

### Protocol 3

#### Summary of original data

- The original data presented is qualitative (images of Western blots). We used Image Studio Lite (LICOR) to perform densitometric analysis of the presented bands. We then performed a priori power calculations with a range of assumed standard deviations to determine the number of replicates to perform.
- Note: band intensity quantified from the image reported in Figure 3A:

**Table:** 

Target	Myc-eptitope tagged vector	Drug	Band intensity normalized to IP myc	Assumed N
BRAF	CRAF	885-A	0.01904	3
		DMSO	0.94756	3
	R89L	885-A	0.27776	3
		DMSO	0.65427	3

#### Test family

- Two-way ANOVA (2 x 2) on BRAF values followed by Bonferroni corrected comparisons for the following groups:
  - Compare the band intensity of BRAF in myc-tagged CRAF^WT^ or CRAF^R89L^ in cells treated with or without 885-A

#### Power calculations

- Power calculations were performed using R software version 3.1.2 (R Core Team, 2014) and G*Power (version 3.1.7) (Faul et al., 2007)
- 2% variance:
  - ANOVA: Fixed effects, special, main effects, and interactions; alpha error = 0.05

**Table:** 

Groups	F test statistic	Partial eta^2^	Effect size *f*	A priori power	Total sample size
myc-tagged CRAF^WT^ or CRAF^R89L^in cells with or without 885-A	F_(1.8)_ = 1628.39 (interaction)	0.9951	14.267	98.7%	5 (4 groups)

- Bonferroni- corrected planned comparisons; alpha error = 0.025

**Table:** 

Group 1	Group 2	Effect size d	A priori power	Sample size per group
CRAF +885A	CRAF +DMSO	69.2756	99.9%	2
R89L +885A	R89L +DMSO	37.4562	99.9%	2

- 15% variance:
  - ANOVA: Fixed effects, special, main effects, and interactions; alpha error = 0.05

**Table:** 

Groups	F test statistic	Partial eta^2^	Effect size *f*	A priori power	Total sample size
myc-tagged CRAF^WT^ or CRAF^R89L^in cells with or without 885-A	F_(1.8)_ = 28.9491 interaction	0.7835	1.9023	90.2%	7 (4 groups)

- Bonferroni- corrected planned comparisons; alpha error = 0.025

**Table:** 

Group 1	Group 2	Effect size d	A priori power	Sample size per group
CRAF +885A	CRAF +DMSO	9.2367	88.1%	2
R89L +885A	R89L +DMSO	4.9941	96.0%	3

- 28% variance:
  - ANOVA: Fixed effects, special, main effects, and interactions; alpha error = 0.05

**Table:** 

Groups	F test statistic	Partial eta^2^	Effect size *f*	A priori power	Total sample size
myc-tagged CRAF^WT^ or CRAF^R89L^in cells with or without SB590885	F_(1.8)_ =8.311 interaction	.05094	1.0191	82.5%	11 (4 groups)

- Bonferroni- corrected planned comparisons; alpha error = 0.025

**Table:** 

Group 1	Group 2	Effect size d	A priori power	Sample size per group
CRAF +885A	CRAF +DMSO	4.9482	95.8%	3
R89L +885A	R89L +DMSO	2.6754	90.1%	5

- 40% variance:
  - ANOVA: Fixed effects, special, main effects, and interactions; alpha error = 0.05

**Table:** 

Groups	F test statistic	Partial eta^2^	Effect size *f*	A priori power	Total sample size
myc-tagged CRAF^WT^ or CRAF^R89L^in cells with or without SB590885	F_(1.8)_ = 4.071 interaction	0.3372	0.7133	80.3%	18 (4 groups)

- Bonferroni- corrected planned comparisons; alpha error = 0.025

**Table:** 

Group 1	Group 2	Effect size d	A priori power	Sample size per group
CRAF +885A	CRAF +DMSO	3.4638	94.0%	4
R89L +885A	R89L +DMSO	1.8728	81.2%	7

- Based on these power calculations, we will run the experiment three times. Each time, we will quantify band intensity. We will determine the standard deviation of band intensity across the biological replicates and combine this with the effect size from the original study to calculate the number of replicates necessary to reach a power of at least 80%. We will then perform additional replicates, if required, to ensure the experiment has more than 80% power to detect the original effect.

### Protocol 4

#### Summary of original data

- The original data presented is qualitative (images of Western blots). We used Image Studio Lite (LICOR) to perform densitometric analysis of the presented bands. We then performed a priori power calculations with a range of assumed standard deviations to determine the number of replicates to perform.
- Note: band intensity quantified from the image reported in Figure 3B:

**Table:** 

Target	Myc-eptitope tagged vector	Drug	Band intensity normalized to IP myc	Assumed N
CRAF	BRAF	885-A	0.0320	3
		DMSO	0.6015	3
	R188L	885-A	0.0164	3
		DMSO	0.1012	3

#### Test family

- Two-way ANOVA (2 x 2) on CRAF values followed by Bonferroni corrected comparisons for the following groups:
  - Compare the band intensity of BRAF in myc-tagged BRAF^WT^ or BRAF^R188L^ in cells treated with or without 885-A

#### Power calculations

- Power calculations were performed using R software version 3.1.2 (R Core Team, 2014) and G*Power (version 3.1.7) (Faul et al., 2007)
- 2% variance:
  - ANOVA: Fixed effects, special, main effects, and interactions; alpha error = 0.05

**Table:** 

Groups	F test statistic	Partial eta^2^	Effect size *f*	A priori power	Total sample size
myc-tagged BRAF^WT^ or BRAF^R188L^in cells with or without 885-A	F_(1.8)_ = 4718.4 (interaction)	0.998	24.28	99.9%	5

- Bonferroni- corrected planned comparisons; alpha error = 0.025

**Table:** 

Group 1	Group 2	Effect size d	A priori power	Sample size per group
BRAF +885A	BRAF +DMSO	66.85	99.9%	2
R188L +885A	R188L +DMSO	58.51	99.9%	2

- 15% variance:
  - ANOVA: Fixed effects, special, main effects, and interactions; alpha error = 0.05

**Table:** 

Groups	F test statistic	Partial eta^2^	Effect size *f*	A priori power	Total sample size
myc-tagged BRAF^WT^ or BRAF^R188L^in cells with or without 885-A	F_(1.8)_ = 83.88 interaction	0.913	3.238	95.6%	6

- Bonferroni- corrected planned comparisons; alpha error = 0.025

**Table:** 

Group 1	Group 2	Effect size d	A priori power	Sample size per group
BRAF +885A	BRAF +DMSO	8.914	86.3%	2
R188L +885A	R188L +DMSO	7.801	99.9%	3

- 28% variance:
  - ANOVA: Fixed effects, special, main effects, and interactions; alpha error = 0.05

**Table:** 

Groups	F test statistic	Partial eta^2^	Effect size *f*	A priori power	Total sample size
myc-tagged BRAF^WT^ or BRAF^R188L^in cells with or without 885-A	F_(1.8)_ = 24.07 interaction	0.750	1.734	85.0%	7

- Bonferroni- corrected planned comparisons; alpha error = 0.025

**Table:** 

Group 1	Group 2	Effect size d	A priori power	Sample size per group
BRAF +885A	BRAF +DMSO	4.775	94.5%	3
R188L +885A	R188L +DMSO	4.179	87.8%	3

- 40% variance:
  - ANOVA: Fixed effects, special, main effects, and interactions; alpha error = 0.05

**Table:** 

Groups	F test statistic	Partial eta^2^	Effect size *f*	A priori power	Total sample size
myc-tagged BRAF^WT^ or BRAF^R188L^in cells with or without 885-A	F_(1.8)_ = 11.79 interaction	0.596	1.214	82.7%	9

- Bonferroni- corrected planned comparisons; alpha error = 0.025

**Table:** 

Group 1	Group 2	Effect size d	A priori power	Sample size per group
BRAF +885A	BRAF +DMSO	3.343	92.3%	4
R188L +885A	R188L +DMSO	2.925	83.9%	4

- Based on these power calculations, we will run the experiment three times. Each time, we will quantify band intensity. We will determine the standard deviation of band intensity across the biological replicates and combine this with the effect size from the original study to calculate the number of replicates necessary to reach a power of at least 80%. We will then perform additional replicates, if required, to ensure the experiment has more than 80% power to detect the original effect.

### Protocol 5

#### Summary of original data

- The original data presented is qualitative (images of Western blots). We used Image Studio Lite (LICOR) to perform densitometric analysis of the presented bands. We then performed a priori power calculations with a range of assumed standard deviations to determine the number of replicates to perform.
- Note: band intensity quantified from the image reported in Figure 4D
  - The band intensities for two groups were beyond the dynamic range for intensity calculation:
    - IP myc-tagged BRAF in cells transfected with the BRAF mutant (D594A): In this case, we used the value for band intensity of IP myc-tagged BRAF in cells transfected with wild type BRAF as an estimate. Since the band for wild type BRAF transfected cells was less intense, this underestimates the effect size, so we are likely overestimating the sample size required.

**Table:** 

Target	Vector	Band intensity normalized to IP myc	Assumed N
IP CRAF	BRAF	0.164	3
	D594A	0.739	3

#### Test family

- Unpaired two-tailed Welch’s *t*-test, alpha error = 0.05.

#### Power calculations

- Power calculations were performed using R software version 3.2.2 (R Core Team, 2014)

**Table:** 

Group 1	Group 2	Variance estimate	Effect size (Glass’ ∆)^1^	A priori power	Sample size per group
BRAF^WT^	BRAF^D594A^	2%	175.30	>99.9%	2
		15%	23.374	89.9%	2
		28%	12.522	93.3%	3
		40%	8.7652	90.8%	4

^1^ The BRAF group SD was used as the divisor.

- Based on these power calculations, we will run the experiment three times. Each time, we will quantify band intensity. We will determine the standard deviation of band intensity across the biological replicates and combine this with the effect size from the original study to calculate the number of replicates necessary to reach a power of at least 80%. We will then perform additional replicates, if required, to ensure the experiment has more than 80% power to detect the original effect.
